# Effect of Varying Expression of EpCAM on the Efficiency of CTCs Detection by SERS-Based Immunomagnetic Optofluidic Device

**DOI:** 10.3390/cancers12113315

**Published:** 2020-11-10

**Authors:** Marta Czaplicka, Krzysztof Niciński, Ariadna Nowicka, Tomasz Szymborski, Izabela Chmielewska, Joanna Trzcińska-Danielewicz, Agnieszka Girstun, Agnieszka Kamińska

**Affiliations:** 1 Institute of Physical Chemistry, Polish Academy of Sciences, Kasprzaka 44/52, 01-224 Warsaw, Poland; mczaplicka@ichf.edu.pl (M.C.); knicinski@ichf.edu.pl (K.N.); anowicka@ichf.edu.pl (A.N.); tszymborski@ichf.edu.pl (T.S.); 2 Department of Pneumology, Oncology and Allergology, Medical University of Lublin, Jaczewskiego 8, 20-950 Lublin, Poland; izachm@wp.pl; 3 Department of Molecular Biology, Institute of Biochemistry, Faculty of Biology, University of Warsaw, Miecznikowa 1, 02-096 Warsaw, Poland; jtd@biol.uw.edu.pl (J.T.-D.); agirstun@biol.uw.edu.pl (A.G.)

**Keywords:** microfluidic device, surface-enhanced Raman spectroscopy (SERS), magnetic nanoparticles, circulating tumor cells (CTCs), cancer, Fe_3_O_4_@Ag@, human metastatic prostate adenocarcinoma cells (LNCaP), human lung carcinoma cells (A549), human prostate adenocarcinoma cells (PC3), cervical cancer cells (HeLa)

## Abstract

**Simple Summary:**

In this work we present a magnetically supported SERS-based immunoassay based on solid SERS-active support for the detection of circulating tumor cells. The SERS response in our optofluidic device was correlated with the level of EpCAM expression. The level of EpCAM cell expression in four cell lines with relatively high (human metastatic prostate adenocarcinoma cells (LNCaP)), medium (human metastatic prostate adenocarcinoma cells (LNCaP)), weak (human metastatic prostate adenocarcinoma cells (LNCaP)), and no EpCAM expressions (cervical cancer cells (HeLa) has been estimated using Western Blot method supported by immunochemistry and correlated with responses of immunomagnetic SERS-based analysis. The capture efficiency of developed assay was investigated in metastatic lung cancer patients. The assay demonstrates the capability to detect circulating tumor cells from blood samples over a broad linear range (from 1 to 100 cells/mL) reflecting clinically relevant amount of CTCs depending on the stage of metastasis, age, applied therapy.

**Abstract:**

The circulating tumor cells (CTCs) isolation and characterization has a great potential for non-invasive biopsy. In the present research, the surface–enhanced Raman spectroscopy (SERS)-based assay utilizing magnetic nanoparticles and solid SERS-active support integrated in the external field assisted microfluidic device was designed for efficient isolation of CTCs from blood samples. Magnetic nanospheres (Fe_2_O_3_) were coated with SERS-active metal and then modified with *p*-mercaptobenzoic acid (*p*-MBA) which works simultaneously as a Raman reporter and linker to an antiepithelial-cell-adhesion-molecule (anti-EpCAM) antibodies. The newly developed laser-induced SERS-active silicon substrate with a very strong enhancement factor (up to 10^8^) and high stability and reproducibility provide the additional extra-enhancement in the sandwich plasmonic configuration of immune assay which finally leads to increase the efficiency of detection. The sensitive immune recognition of cancer cells is assisted by the introducing of the controllable external magnetic field into the microfluidic chip. Moreover, the integration of the SERS-active platform and *p*-MBA-labeled immuno-Ag@Fe_2_O_3_ nanostructures with microfluidic device offers less sample and analytes demand, precise operation, increase reproducibly of spectral responses, and enables miniaturization and portability of the presented approach. In this work, we have also investigated the effect of varying expression of the EpCAM established by the Western Blot method supported by immunochemistry on the efficiency of CTCs’ detection with the developed SERS method. We used four target cancer cell lines with relatively high (human metastatic prostate adenocarcinoma cells (LNCaP)), medium (human metastatic prostate adenocarcinoma cells (LNCaP)), weak (human metastatic prostate adenocarcinoma cells (LNCaP)), and no EpCAM expressions (cervical cancer cells (HeLa)) to estimate the limits of detection based on constructed calibration curves. Finally, blood samples from lung cancer patients were used to validate the efficiency of the developed method in clinical trials.

## 1. Introduction

Circulating tumor cells (CTCs) are cancerous cells that have broken away from the original tumor and once circulating with other cells in the peripheral blood cause metastasis. Clinical research results have demonstrated that the metastasis lead to over 90% of tumor-related deaths [1]. Therefore, the CTCs are clinically important biomarkers for monitoring, prognosis of cancer therapy, and efficient treatment of patients [2,3,4,5]. Isolation of CTCs from blood is still a challenging task owing to their extreme rarity, typically 1–10 CTCs in 1 mL blood sample [6,7], and the heterogeneity of their surface markers [8]. Over the last two decades, various CTC separation methods utilizing physical and biochemical properties of cancer cells have been proposed by both research groups and companies [9,10,11], The label-dependent (biological) methods include the immunocapture [12,13,14,15,16,17], immunomagnetophoresis [18,19,20,21,22], and immunofluorescence [23,24] approaches. The physical, label-free methods are mainly based on the fact that CTCs are usually larger than normal blood cells [25]. The mechanical filtration [21,26,27,28,29], dielectrophoresis [30,31,32], acoustophoresis [33,34,35], and hydrodynamic [36,37] approaches enable the efficient isolation of CTCs from whole blood samples. Although some label-independent technologies for CTCs’ isolation have satisfactory performance, they still have several limitations. The main drawback is their low specificity, due to the purity, recovery rate, and viability, as the results of the overlap in size between CTCs and normal blood cells [38] damage and clog trapped CTCs in the filtrating materials [39]. Conversely, the label-dependent method for both positive and negative CTCs’ isolation advanced label-free technologies in terms of specificity and purity [40,41,42]. This method might also be limited by low throughput due to little or no expression of cancer-specific surface markers [43]. However, taking into account the high efficiency of their specificity and sensitivity, these label-dependent methods are most commonly used in the research laboratory and also have been adopted in commercial technologies for CTC isolation [44,45,46,47]. The immunocapture approaches, that based on binding epithelial-specific surface markers (e.g., EpCAM, HER2, and MUC1) [12,13,48] and using leucocyte-specific markers (e.g., CD 45 and CD66b) [10,14] are mostly adopted for label-dependent CTCs’ isolation. The protocol of these methods often involve complex sample preparation and require several separation techniques for CTCs’ filtration, enrichment, numeration, and analysis.

It should be also noticed, that the commercial CellSearch system (Veridex LLC) is the current ‘gold standard method’ permitting the CTCs’ detection and enumeration and was used for the analysis of CTCs in metastatic breast [4], prostate [49], and colon cancer patients [50].

Although the accuracy of immunofluorescence is well established, this technique still suffers from well-known drawbacks: quenching of the fluorescence signals at excitation, many false positives caused by nonspecific absorption of antibodies, and the broad emission profiles of fluorophores leading to bands overlapping, thus, limiting the level of multiplexing quantitative analysis.

Over recent years, the surface–enhanced Raman spectroscopy (SERS) has been increasingly applied in non-invasive cancer diagnostic studies, including CTCs [51,52,53,54,55,56], cancer-related protein [57,58,59], exosomes [60,61,62], circulating tumor DNAs [63,64,65], or micro-RNA [66,67,68,69].

Raman spectroscopy is a light scattering method, used to measure the vibrational energy modes of a samples. The method can provide a molecular fingerprint of a sample, but the signals have very poor efficiency of Raman scattering and have strong background fluorescence. When the analyte is adsorbed on the rough surface of metal (usually made of silver and gold) [70], we can observe an increase of the weak Raman signal, known as surface-enhancement Raman spectroscopy and was first reported by Fleishman et al. in 1974 [71].

The SERS-based immunoassay offers a higher sensitivity that other clinically used detection techniques [72] and enables detection of simulations of multiple biomarkers in complex biological fluids [65,73]. Sha et al. [55], for the first time, adapted the SERS technique for the detection of CTCs in human blood. Ten years later, in 2018, Pang et al. [51] presented the SERS biosensor for the detection of hepatocellular carcinoma (HCC) circulating tumor cells using developed dual-enhanced SERS nanoprobes. In the same year, Tsao et al. [74] demonstrated the use of antibody-conjugated and Raman reporter-coated gold nanoparticles for multiplex CTC surface marker monitoring. In all of these approaches, the suitable modified metallic nanoparticles with antibodies/aptamers and/or Raman reporters were used to generate the SERS signals. The SERS effect in nanoparticles is associated with the excitation of surface plasmon resonances by the incited laser. In fact, the magnitude of SERS enhancement is associated with local field “hot spots” between aggregated metallic nanoparticles [75] and lead to huge irreproducibility of recorded signals. The enhancement level fluctuates from particle to particle and is strongly related with their aggregation and environment properties.

In this work, we present for the first time, a magnetically supported SERS-based immunoassay based onto solid SERS-active support for the detection of circulating tumor cells. The SERS-active solid support plays an important role in the sensitivity of SERS-based immunoassay. It was found that enhanced electromagnetic field is not only excited around the gold or silver nanoparticles, but is also generated on the SERS-active substrate [76]. We have developed a sensitive SERS substrate based on the femtosecond laser induced silicon which possesses a very strong enhancement factor (up to 10^8^), high stability and reproducibility, which satisfies all spectroscopic features required for bioanalytical applications. The SERS surfaces were prepared based on a simple protocol according to patent application [77]. Additionally, we have designed SERS-immune probes (the Raman reporter-labeled–immune-magnetic nanoparticles; anti-EpCAM/Ag@Fe_2_O_3_/*p*-MBA) which demonstrate spectroscopic, magnetic, and immune performance. Epithelial cell adhesion molecule (EpCAM or murine (CD326)) is a trans-membrane glycoprotein, consisting of 314 amino acids [78]. Its main function is contribution in cell-cell interaction, but it also plays important roles in cell differentiation signaling, migration, and proliferation [79].

EpCAM was originally recognized in colon cancer in 1979 and recently is known as a highly expressed antigen on a variety of carcinomas, thus, it is utilized as a carcinoma marker [80]. As the EpCAM is highly expressed on normal epithelia and epithelial cancer cells and inattentive on blood cells it is commonly used for capturing of epithelial CTCs.

The combined *p*-MBA-labeled immuno-Ag@Fe_2_O_3_ nanostructures comprised of: (i) iron(III) oxide (Fe_2_O_3_) covered with SERS-active metal (Ag), (ii) a sub-monolayer of reporter molecules (*p*-mercaptobenzoic acid, *p*-MBA) chemisorbed onto Ag, and (iii) the antibody (anti-EpCAM antibody) covalently bound to a thiol layer. The application of an external magnetic field induced the directing flow of SERS-immune probes to SERS-active which lead to the efficient and sensitive immune recognition of cancer cells. The magnetic field preserved also the stability of the formed immune complexes at each step of procedure, from CTCs’ enrichment and washing to their identification.

The integration of SERS-active platform and *p*-MBA-labeled immuno-Ag@Fe_2_O_3_ nanostructures with the chip ensures less sample and analyte demand, precise operation, increase reproducibly of spectral responses and, enables miniaturization and portability of presented approach. Additionally, it should be highlighted that so-far presented SERS-immunoassays utilized a wide range of silver and gold nanoparticles with different sizes, shapes, and modification arrangements [81,82,83,84,85]. In such an immunoassay, the enhancement effect and the sensitivity of immune recognition depend mainly on the surface plasmon resonance of the modified Ag or Au nanoparticles. In our approach, we also adopted the solid SERS-active platform based onto femtosecond laser induced silicon to generate the extra-enhancement for analytes preset in such a sandwich plasmonic arrangement.

The level of EpCAM cell expression in four cell lines, i.e., LNCaP, PC3, A549, and HeLa, has been estimated using immunocytochemistry and correlated with responses of immunomagnetic SERS-based analysis. The capture efficiency of developed assay was investigated in metastatic lung cancer patients. The results indicate that at lower EpCAM expression levels the quantitative capturing all CTCs in a blood sample may be problematic, especially decrease the utility of surface proteins based methods.

## 2. Experimental Section

### 2.1. Cells Cultivation and Preparation of Cell Suspensions

All the cell lines (A549, HeLa, LNCaP, and PC3) came from the European Collection of Cell Cultures (ECACC) and were supplied by Sigma-Aldrich (St. Louis, MO, USA).

Cells were cultured in the following media: (i) A549 cells in F-12K medium (Kaighn’s modification of Ham’s F-12, ATCC, Manassas, VA, USA), (ii) HeLa cells in Dulbecco’s modified Eagle’s medium (DMEM), (iii) LNCaP cells in RPMI 1640 (Biowest, Nuaillé, France) medium supplemented with 10 mM HEPES (4-(2-Hydroxyethyl)piperazine-1-ethanesulfonic acid), 1 mM sodium pyruvate and glucose up to 4.5 g/L, (iv) PC3 cells in RPMI-1640 medium. All media were supplemented with 10% FBS, streptomycin (100 μg/mL) and penicillin (100 U/mL). The cell cultures were:

(i). cultured in humidified atmosphere of 5% CO_2_ at the 37 °C as the optimal temperature;
(ii). trypsinized (0.05% trypsin, 0.02% EDTA (Ethylenediaminetetraacetic acid) solution);
(iii). washed with PBS buffer to reach subconfluency (about 80%);
(iv). finally, cells were collected by centrifugation for 5 min at room temperature at 250× *g*. The cell pellets were re-suspended in PBS to reach the concentration ca. 0.44 × 10^6^ cells/mL and stored on ice.

For SERS analysis, the cells were counted manually and subsequently added to healthy volunteer blood samples. The 2 mL of blood and 80 μL of modified nanoparticles at the final concentration 0.2 mg/mL were used for the particular experiment.

### 2.2. Western Blotting of Cell Lysates

**Figure 1:**
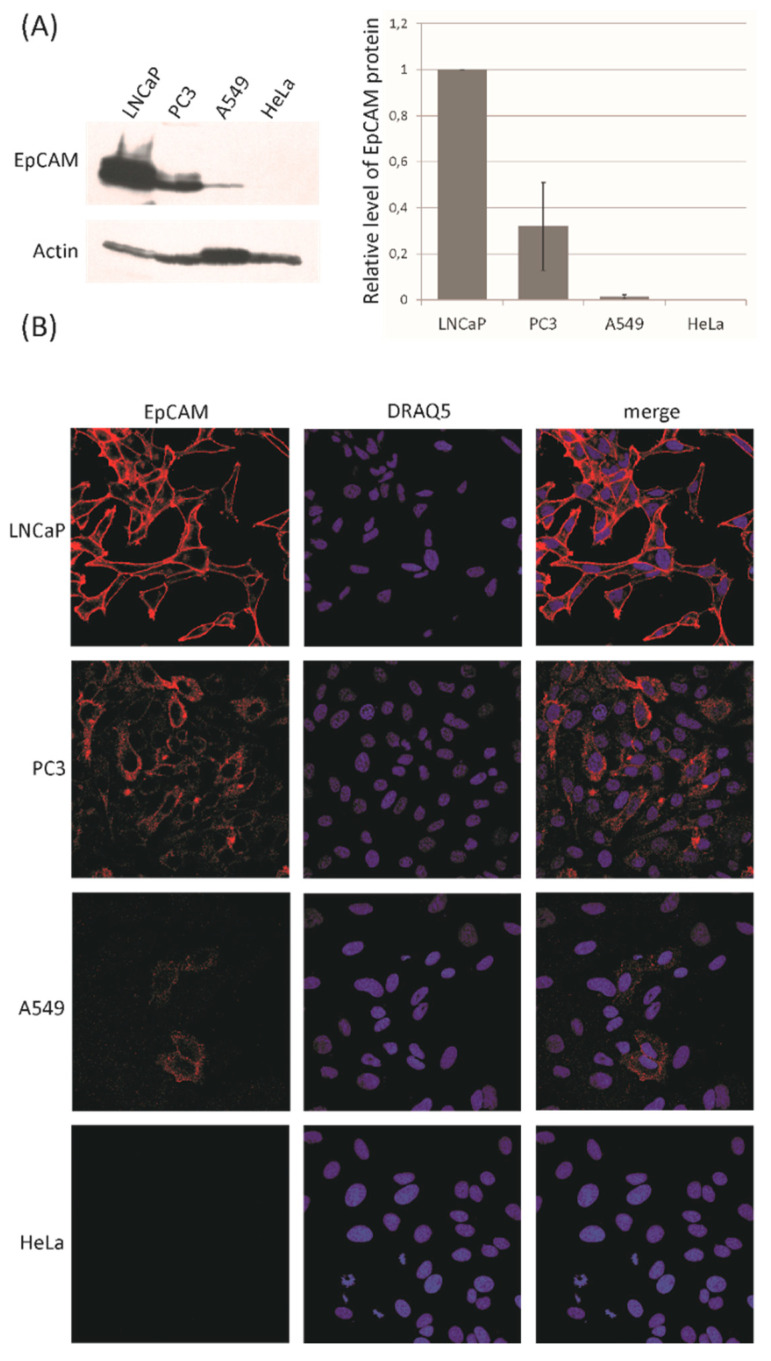
Epithelial-cell-adhesion-molecule (EpCAM) expression in selected cancer cell lines. (**A**) Whole cell lysates were analyzed by Western blotting with anti-EpCAM and anti-actin antibodies (3 independent experiments). The level of Actin protein was chosen as a loading control. The bar graph shows the densitometric quantification of the Western blots and it presents the relative expression of EpCAM protein in reference to the level of EpCAM in human metastatic prostate adenocarcinoma cells (LNCaP) cells (arbitrarily set to 1; *n* = 3, ±SD). (**B**) Immunocytochemical analysis was performed with anti-EpCAM antibodies. EpCAM protein was detected in LNCaP, human prostate adenocarcinoma cells (PC3), and human lung carcinoma cells (A549) but not in cervical cancer cells (HeLa) cells (red, left panel). Nuclear counterstaining was performed using DRAQ5™ fluorescent probe (blue, middle). Merged images are shown in the right panel. All images were obtained with Plan Apochromat VC 60×/1.40 oil DIC objective and full fields of view are shown. Representative confocal images are presented in each case. The bar graph presents a relative expression of EpCAM calculated from densitometric measurements of three independent Western blot analysis. In each analysis the level of EpCAM expression was arbitrarily set to 1, therefore the error bar is actually equal to 0 in this case. Additional data for the Western blot analysis shown in Figure 1 are included in Supplementary Materials (Figure S5).

For Western blotting analysis cells were lysed by boiling in the Laemmli buffer. Cell pellets were re-suspended in the Laemmli buffer to reach the concentration of 1 × 10^4^ cells per 1 μL and boiled for 20 min. Samples corresponding to 2 × 10^5^ cells were separated by standard SDS-PAGE followed by electrotransfer of proteins onto the PVDF (Poly(vinylidene fluoride)) membrane (Bio-Rad, Hercules, CA, USA). Immunodetection of the proteins was carried out using following primary antibodies: (i) rabbit anti-EpCAM [EPR20532-225] IgG (ab223582, Abcam, Cambridge, UK) and (ii) mouse anti-actin IgM (SAB4200248; Sigma-Aldrich, St. Louis, MO, USA). Secondary anti-rabbit IgG-HRP (SC2030; Santa Cruz, Dallas, TX, USA) were used in case of anti-EpCAM primary antibodies and anti-mouse IgM-HRP (ADI-SAB-110, Enzo, Farmingdale, NY, USA) in case of anti-actin primary antibodies. Densitometric analysis of the Western blots were performed using QuantityOne software (Bio-Rad, Hercules, CA, USA). Actin protein level was used as a control of the equal application of lysates. Quantification for EpCAM, presented in Figure 1A and in Table 1 was adjusted based on the amount of actin in each sample to compensate for differences in the total amount of cellular proteins loaded on the electrophoretic gel.

### 2.3. Immunocytochemistry

For immunocytochemical analysis A549, HeLa, LNCaP, and PC3 cells were cultivated on coverslips and subsequently fixed with 4% paraformaldehyde, permeabilized with 0.1% Triton X-100 in PBS, blocked with PBS containing 3% bovine serum albumin and subjected to staining with primary antibodies (rabbit anti-EpCAM [EPR20532-225] and rabbit anti-EpCAM [7504], IgG (ab223582, Abcam)) followed by secondary antibodies (goat anti-rabbit IgG, Alexa Fluor 594, A11012 Invitrogen/Thermo Fisher Scientific, Waltham, MA, USA). Finally, cell nuclei were stained with DRAQ5™ fluorescent probe (Biostatus, Shepshed, UK) and coverslips were mounted with Dako fluorescent mounting medium (Dako North America, Carpinteria, CA, USA). The stained cells were observed under Nikon A1R MP confocal laser scanning microscope (Nikon Instruments, Melville, NY, USA) equipped with a Plan Apochromat VC 60×/1.40 oil DIC objective. Obtained images were proceeded with NIS Elements software (Nikon Instruments, Melville, NY, USA). To exclude non-specific labelling by secondary antibodies, control cells were incubated without the primary antibodies. Under negative control conditions no immunostaining was observed for any of the analyzed cell lines.

### 2.4. Immunoassay Protocol

The SERS surfaces were prepared based on a simple protocol according to patent application [77]. The coated samples were immersed in a solution of folic acid (Sigma-Aldrich) in ethanol, concentration was 0.1 mM for 24 h to modify the surface. In the next step, platforms were modified by EDC/NHS standard procedure to form an amide linkage (EDC = 1-ethylo-3-(3-dimethyloaminopropyl)carbodiimide and NHS-hydroxysulfosuccinimide). The 12.5 µL of 0.2 M EDC (Sigma-Aldrich) and 12.5 µL of 0.05 M NHS (Sigma-Aldrich) mixture in deionized water have been applied to the substrate. After 45 min the 50 µL of 1 mg/mL anti-EpCAM antibody (Abcam ab7504) was added. The modified samples were allowed and incubated for 3 h.

### 2.5. Materials and Reagents

#### 2.5.1. Design and Fabrication of a Microfluidic Chip

The magnetically controlled microfluidic chip integrated with SERS-active platform is presented in Scheme S1 (Supplementary Materials). The microfluidic device was designed using CAM software (MasterCAM, CNC Software, Inc., Tolland, CT, USA). Afterwards, the channels were micromachined with a computer numerical-controlled (CNC) milling machine (ErgWind, Poland). The chip was made using polycarbonate plates (Bayer, Leverkusen, Germany) with a thickness of 3 and 5 mm.

#### 2.5.2. Lung Cancer Patients

Peripheral blood samples were drawn by venipuncture into 9 mL S-Monovette^®^ Hematology EDTA K3 pre-dosed as a liquid preparation in an average concentration of 1.6 mg EDTA/mL blood. The maximum dilution caused by the liquid preparation is lower than 1%. Further procedures were performed directly. Blood samples were obtained from healthy donors and metastatic lung cancer patients treated at the Department of Pulmonology, Allergology and Oncology at Medical University of Lublin, Poland. All patients have been diagnosed with stage IV of non-small cell lung cancer including adenocarcinoma and squamous histology. The probability of cancer cell in the peripheral blood was high due to high burden of the disease. Data were collected for age, ethnicity, histological subtype, stage of disease, smoking status, sites of metastases, and treatment received.

The studies where completed in agreement with relevant regulations. All patients provided written informed consent and the study protocol was approved by the medical ethical committee of the University Medical of Lublin, Poland (KE-0254/95/2018).

#### 2.5.3. Preparation of Ag@Fe_2_O_3_ Magnetic Nanoparticles

The route for synthesis the Ag@Fe_2_O_3_ nanoparticles was modified compared to that previously reported by G. Sharma and P. Jeevanandam [86]. This approach is a simple one step, thermal decomposition of silver salt in the presence of iron oxide nanospheres. Fe_2_O_3_ nanoparticles with a diameter less than 50 nm (purchased from Sigma-Aldrich) were pre-grounded in a ceramic mortar with silver acetate (purchased from Sigma-Aldrich) in a mass ratio of 1:100. The pre-homogenized mixture was transferred to plastic containers in which agate milling balls were placed. Then the mixture was grounded in a Retsch MM400 mixer ball mill with a frequency of 20 Hz for 30 min. The mixture was then transferred to a round bottom boat crucible and placed in a wire wound Carbolite tube furnace. The contents were heated to 200 °C with heating rate of 10 °C/min and kept under a nitrogen atmosphere for two hours and finally cooled down to ambient temperature in a protective nitrogen atmosphere. In the next stage, the product was purified by alternately suspending it in new portion of 500 mL of distilled water using ultrasonic bath for this process and pouring the suspension over neodymium magnet till the supernatant became clear. In this way, the non-magnetic phase and residuals from synthesis remained suspended and dissolved in distilled water. The remaining product was dried at ambient temperature and ready for further modifications.

#### 2.5.4. Preparation of the Raman Reporter-Labeled-Immune-Magnetic Nanoparticles (anti-EpCAM/Ag@Fe_2_O_3_/p-MBA)

**Figure 2:**
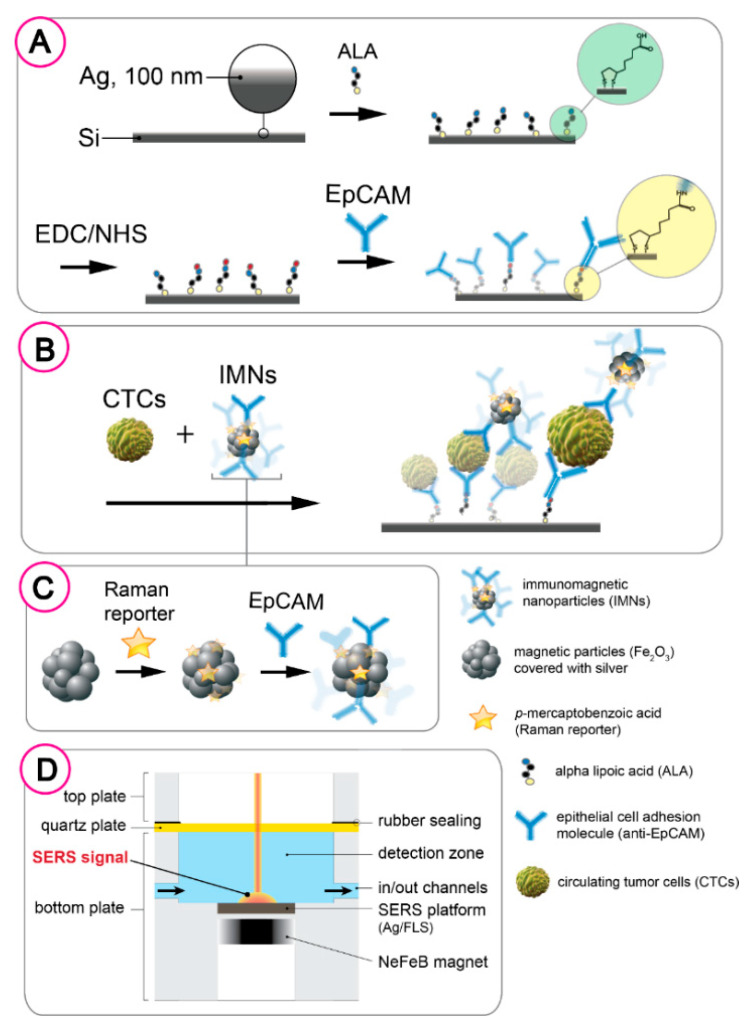
Structure of magnetically-supported surface-enhanced Raman spectroscopy (SERS)-based analysis of CTCs. (**A**) SERS platform modification with folic acid. (**B**) Anti-EpCAM antibodies capturing on the modified SERS platform via EDC/NHS (1-ethylo-3-(3-dimethyloaminopropyl)carbodiimide/hydroxysulfosuccinimide) strategy. (**C**) Addition of target CTC and Raman reporter-labeled–immune-magnetic nanoparticles to form immunocomplexes. (**D**) Detection zone (DZ) chamber.

For the acid (*p*-MBA) that adsorbed on silver coated magnetic nanoparticles via thiol group was applied as a Raman reporter. The 10^−3^ M ethanol solution of *p*-MBA was prepared. In the next step the *p*-MBA-labeled–immune-magnetic nanoparticles (Figure 2A) were prepared by conjugate an epithelial cell adhesion molecule (anti-EpCAM) using EDC/NHS chemistry. In the first step, the carboxyl-group of *p*-MBA immobilized onto magnetic nanoparticles were activated by theirs 3 h incubation in the PBS solution containing mixture of 12.5 mM NHS and 12.5 mM EDC, the 50 µL of 1 mg/mL anti-EpCAM (Abcam ab7504) was added and incubated ca. 4.5 h to complete the conjugation reaction.

#### 2.5.5. Fabrication and Modification of SERS-Active Silicon Substrate

The SERS-active silicon substrate was prepared within three steps:

(i). physical modification of the surface with femtosecond laser,
(ii). sputtering of SERS-active metal layer, and
(iii). chemical modification of the surface.

In the first step, the silicon wafer was mechanically cut into a 3 mm × 3 mm fragments and subjected to laser ablation with femtosecond laser. Detailed information about the procedure and optimization were described in patent application [77]. The femtosecond laser (λ = 1030 nm) is based on a compact fiber chirped-pulse amplification system set in a single pass configuration. The active medium of the oscillator was doped ytterbium crystal (Yb:KYW). The working parameters were: repetition rate 300 kHz, pulse width 300 femtosecond, distance between scanning lines 30 μm and scanning rate of the laser beam on the surface of silicon was 1.5 m/s. The surface was modified with two ablation layers, where scanning layers are mutually perpendicular.

The second step was sputtering of thin layer of SERS-active on the silicon submitted to laser ablation. The PVD device (Quorum, Q150T ES, Laughton, UK) was applied to sputter layer via magnetron sputtering of silver directly on the surface of modified silicon. No adhesion layer, i.e., chromium or titanium, was placed on the surface of silicon before sputtering of silver. The thickness of the silver layer was set to 100 nm for all analyzed samples. The thickness of layer of silver was measured during the process with build-in quartz microbalance. The sputtering layer was applied to the silicon with current 25 mA and under the pressure 10^−2^ mbar. SERS platforms were stored in sterile Petri dish under ambient conditions prior the use. The silver coated femtosecond laser induced SERS-active silicon substrate was incorporated in the chamber of microfluidic chip (Figure 2D).

Finally, this SERS-active platform was modified with 1 mM ethanoic solution of lipoic acid for 24 h to achieve the carboxyl-terminated linkage layer. Afterwards, the thiol-modified surface was completed by injecting the mixture of 12.5 µL of the activation solution (50 mM NHS and 100 mM EDC in the ratio 1:10 in deionized water) and 50 µL/mL anti-EpCAM solution in PBS buffer (pH = 7.2) in the ratio 1:6 into the detection zone of microfluidic chamber (see Figure 2D). After 3 h the antibodies modified SERS platform was washed with 1 mL of PBS buffer solution ran through the microfluidic chamber at flow rate 4.5 mL/min.

## 3. Equipment

### 3.1. Raman and SERS Measurements

Raman and SERS spectra were collected using a Renishaw InVia Reflex spectrometer (Gloucestershire, UK) with instrument control software (Renishaw WiRE 3). The spectrometer is equipped with: (i) 785 nm diode laser and the thermoelectrically cooled (400 × 575 pixels) CCD detector, (ii) Leica DMLM × 50 microscope objective (Numerical Aperture (NA) 0.75).

The spectral range is 3400–200 cm^−1^ with a spectral resolution of 2.5 cm^−1^. Spectra were recorded with the 10 s accumulation of 4 scans. The power of the diode laser was 2.5 mW at the sample. Recorded SERS spectra were exported to the OPUS software package (Bruker Optic GmbH, 2012 version) for the background correction (10 itenary and 64 points) and normalization using a ‘Min-Max normalization’.

### 3.2. SEM Measurements

The morphology of the SERS platform was examined with Scanning Electron Microscopy (SEM). The images were taken with FEI Nova NanoSEM 450 microscope (Hillsboro, OR, USA). The accelerating voltage was set between 5 and 10 kV and the Secondary Electrons (SE) mode was used. The samples were attached to the SEM stabs with a carbon tape and were observed without any additional conductive layer on the surface (e.g., thin layer of gold or carbon).

## 4. Results and Discussion

### 4.1. EpCAM Expression in Selected Cancer Cell Lines

The crucial role of EpCAM in oncogenesis has been extensively studied by many groups [78,87].

It has been reported that it is involved in tumorigenesis, metastasis, and cancer stem cells [88,89].

The prevalence and prognostic significance of circulating tumor cells (CTCs) expressing the cell surface epithelial cell adhesion molecule (EpCAM) in patients with lung, breast and prostate cancer has been also demonstrated [90,91,92,93].

The level of EpCAM expression showed great variation between different cells lines even in the cells belong to the same cancer subtype [94].

Four cell lines were chosen as the model cells for the SERS analysis. The selected cell lines were subsequently used to construct calibration curves to estimate the limits of detection.

According to the Protein Atlas database [95] and published specific data [96] the selected cell lines should differ in the EpCAM expression. To verify the actual expression of EpCAM in the cell populations we cultured, two types of analysis were performed—Western blotting and immunocytochemistry. Densitometric analysis of the Western blots allowed us to calculate the relative level of EpCAM protein in each cell line (Figure 1A). The highest level of EpCAM was observed in LNCaP cells, and it was arbitrarily set to 1. The level of EpCAM in the remaining cell lines was presented as the fraction of the calculated EpCAM level in the LNCaP cells. The analysis showed that the level of EpCAM is moderate in PC3 cells and low in A549 cells. EpCAM protein was not detected in HeLa cells. Immunocytochemistry analysis confirmed the results obtained by Western blot (Figure 1B).

Given the described variability in EpCAM expression of examined four cell lines, we have investigated if and how this could influence the yield of SERS-based immunomagnetic analysis of these cell lines.

### 4.2. SERS-Based Detection Strategy

The procedure of CTC detection is based on the antigen-antibody immune response. As illustrated in Figure 2 the performed immunoassay is consisting of three layers:

(i). the first layer composed of anti-EpCAM antibodies captures on the SERS platform via lipoic acid (LA);
(ii). the second layer is consisted of target cancer cell lines with relatively high (LNCaP), medium (PC3), weak (A549), and no EpCAM expressions (HeLa);
(iii). the third layer is composed of the Raman reporter-labeled–immune-magnetic nanoparticles (anti-EpCAM/Ag@Fe_2_O_3_/*p*-MBA).

The designed microfluidic-SERS chip (Figure S1 and Figure 2) allows us to perform and control the reactions at each step of sandwich (an antibody-antigen-antibody) immune complexes formation and magnetic separation of circulating cancer cells from complex matrix of blood samples. Figure S1 presents a general scheme of immunomagnetic optofluidic device. Immunomagnetic nanoparticles and blood plasma with cancer cells were delivered via syringe pumps into a mixing area of device and then via collective microchannel flow to the detection zone (DZ). The real photo of the microfluidic system connected with syringe pumps is presented in Figure S4.

The detection zone is the chamber with incorporated SERS-active platform, where immune complexes were created and identified via characteristic spectroscopic response. While the fluid with bounded cancer cells reaches the detection zone, a magnetic field is introduced to held them on the antibodies modified SERS-active platform for the formation of sandwich immune structure. Usually, the cancer cells in human blood plasma at the appropriate concentration were injected at the rare 2.5 µL min^−1^ and the Raman reporter-labeled–immune-magnetic nanoparticles (anti-EpCAM/Ag@Fe_2_O_3_/*p*-MBA) were injected into the inlet Y at the rate of 3.5 µL min^−1^. After 3 min the flows were stopped and magnetic field was introduced for 15 min via neodymium permanent magnet (NdFeB) mounted under the detection zone chamber (Figure S1 and Figure 2D). At the end, the PBS buffer solution (pH = 7.2) was injected via inlet remove unbounded magnetic nanoparticles, physically adsorbed cancer cells, and other reagents, that were not magnetically separated from complex fluid matrix.

### 4.3. Capturing Substrate and Characterization of Raman Reporter-Labeled-Immune-Magnetic Nanoparticles

The capturing substrate is based on silicon modified with femtosecond laser and covered with 100 nm layer of silver, which ensures surface with high enhancement factor (EF) and uniformity across the whole substrate. Obtained platform was characterized via SEM to assess the morphology of the silver nanostructures. SEM images (see Figure S2) reveal uniform surface with silver nanostructures of diameter 40 ± 10 nm (based on XRD analysis). Enhancement Factor calculated for the platform and 10^−6^ M *p*-MBA molecules adsorbed on the surface gave EF at a level of 10^8^. The excellent spectral properties of designed plasmonic support (high sensitivity, selectivity and reproducibility of recorded signals) are crucial for bio-medical analysis (see Figure S3).

**Figure 3:**
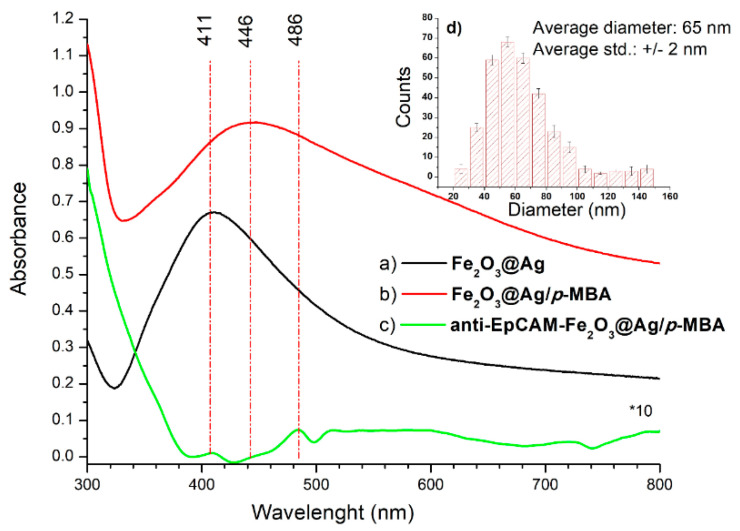
The UV-visible extinction spectra Fe_2_O_3_@Ag magnetic nanoparticles at different steps: (**a**) Fe_2_O_3_@Ag as-received, (**b**) after modification with Raman reporter (*p*-MBA), (**c**) after mixing with antibody (anti-EpCAM antibodies), and (**d**) size-distribution histogram of Fe_2_O_3_@Ag.

**Figure 4:**
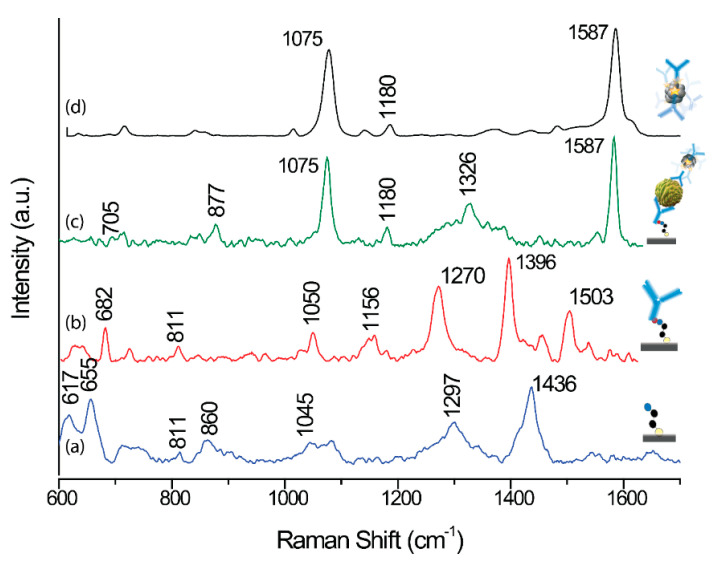
SERS responses in the detection zone (DZ) chamber of microfluidic immunoassay during detection of LNCaP in human blood plasma sample according to the subsequent steps presented in Figure 2: (**a**) SERS spectrum for a monolayer of ALA (alpha lipoic acid) spontaneously adsorbed on femtosecond laser induced SERS-active silicon substrate (Ag/FLs substrate); (**b**) SERS spectrum of ALA after anti-EpCAM antibodies immobilization; (**c**) SERS signals after completed immune reaction: mixed and then delivered target LNCaP cancer cells and anti-EpCAM-Ag@Fe_2_O_3_/*p*-MBA into anti-EpCAM-ALA-Ag/FLs substrate; and (**d**) SERS spectrum of immunomagnetic nanoparticles.

The anti-EpCAM antibodies were covalently bind to the carboxyl-terminated linkage layer of lipoic acid using EDC/NHS coupling method. The formation of the silver–bound thiolates and their subsequent coupling to antibodies was confirmed by Raman spectroscopy (see Figure 4). The successful binding of *p*-MBA and anti-EpCAM antibodies to Fe_2_O_3_@Ag magnetic nanoparticles was confirmed by UV experiments (Figure 3).

As is shown in Figure 3, the as-received Fe_2_O_3_@Ag magnetic nanoparticles have a strong extinction maximum at 411 nm, which is a characteristic peak assigned to pure silver colloid with a diameter <50 nm [97]. Due to the presence of paramagnetic Fe_2_O_3_ in the core of the nanoparticles we observed a blue shift [98] of maximum absorption in comparison to the results presented by Mulfinger et al. [97]. Distribution of the average size of Fe_2_O_3_@Ag, obtained via SEM measurements is ~65 nm. The histogram for these results is presented in Figure 3d. After coating Fe_2_O_3_@Ag magnetic nanoparticles by Raman reporter, the spectrum of Fe_2_O_3_@Ag is red-shifted to 446 nm (Figure 4b), since the LSPR (Localized Surface Plasmon Resonance) band of Fe_2_O_3_@Ag is depending on the immediate molecular surrounding of the particles. The red shift to 486 nm of the surface plasmon resonance peak [99] demonstrates the collective interaction of the electrons of the interconnected particles. After modification with anti-EpCAM antibodies one can observe a large decrease in the strength of this band and the red-shift to 486 nm. This phenomenon [100] indicates bonding of functionalized nanoparticles with anti-EpCAM antibodies (Figure 3c).

These spectral changes in UV-vis spectroscopy indicate that anti-EpCAM antibodies were successfully conjugated onto the surface of Ag@Fe_2_O_3_ magnetic nanoparticles.

### 4.4. SERS Immunomagnetic Detection of CTC in Blood Plasma

Detection and enumeration of CTCs in blood samples is still analytical milestone in cancer diagnostics. In the framework of these studies, we have examined the effect of varying expression of EpCAM in different cell lines on the efficiency of immune-based CTCs recognition. We used four cell lines, as this enabled us to measure the content of cancer cells in the blood sample in our SERS-based device and also to estimate the limit of detection based on constructed calibration curves. Figure 4 presents the representative SERS responses of our immune system onto each step of modifications underlined in the Figure 2.

Figure 4 shows the SERS spectrum for a monolayer of alpha lipoic acid (ALA) spontaneously adsorbed on femtosecond laser induced SERS-active silicon substrate (Ag/FLs substrate). The as-formed ALA monolayer exhibits an intense stretching vibration signal of the adsorbed molecules at 655 and 750 cm^−1^, attributed the S–C–C vibrations [101]. The bands at about 860 cm^−1^ can be associated with the deformation vibrations of the protonated carboxylic group, whereas the well-defined band at 1045 cm^−1^ is due to the ν (C–C) skeletal vibrations. The signal observed at 1295 cm^−1^ is attributed to the CH bending vibration. The strong signal at 1434 cm^−1^ can be associated with symmetrical (νs) vibrations of COOH. The presence of these bands proves the formation of the ALA monolayer with protonated carboxylic group on the Ag/FLs substrate. Upon treatment of the activated via EDC/NHS reaction ALA monolayer with the solution of anti-EpCAM antibodies, (Figure 4b), new bands are observed at 1270, 1396, and 1503 cm^−1^. We assigned these bands to the NH and C=O stretch vibrations in the immobilized antibodies, respectively [102,103]. Figure 4c displays the SERS spectrum recorded in the detection zone of the microfluidic chip (Figure 2D) where immune complexes were created after completing of Ag@Fe_2_O_3_ magnetic nanoparticles and blood plasma with cancer cells. As can be seen, the spectrum is dominated by the most intensive bands at 1075 and 1587 cm^−1^ typical for *p*-MBA [104], which are assigned to ν_8a_ and ν_12_ aromatic ring vibrations, respectively. This characteristic fingerprint spectrum provides a unique code for the capturing the particular target cancer cell. Similarly, strong characteristic SERS signals of *p*-MBA (Figure 4d) were obtained for *p*-MBA immobilized onto the surface of anti-EpCAM-Ag@Fe_2_O_3_ magnetic nanoparticles. Bands appeared in this spectrum at 708, 796, 1075, 1176, 1474, and 1588 cm^−1^ typical for *p*-MBA [104], treated as a fingerprint of this molecule, which indicates that the *p*-MBA can work as an efficient Raman reporter for SERS immunoassay. Table S1 summarizes band assignments for the normal Raman and SERS spectrum of *p*-MBA.

In order to increase the efficiency of detection, the immune recognition was performed: (1) under combination of continuous flow (incubation of SERS probes with blood samples in microfluidic channels) with static steps (immune reaction in the reaction zoon of microfluidic chamber); (2) under introducing the magnetic field to directing separation and enrichment of captured CTCs cells onto modified SERS solid platform.

### 4.5. Quantitative Analysis of Cancer Cells

**Figure 5:**
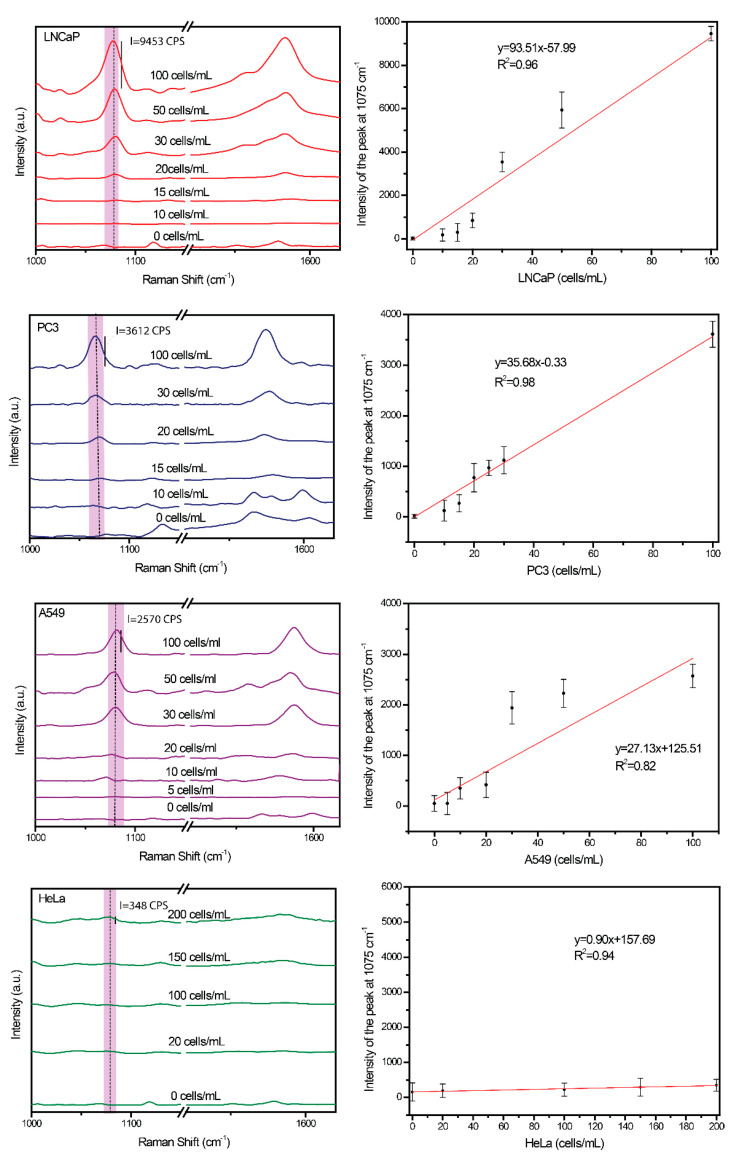
Averaged SERS spectra for LNCaP, PC3, A549, and HeLa in the range from 0 to 100 cells/mL in the peripheral blood, and the corresponding calibration curves.

The capability of the developed SERS-based immunoassay for quantitative analysis of cell lines with different sizes and high (LNCaP), medium (PC3), weak (A549), and no EpCAM expressions (HeLa) was demonstrated. These tumor cells were added to the blood in the manner that simulate the rare circulating tumor cell in real clinical samples in order to construct the calibration curves. Figure 5 presents the recorded SERS spectra for selected concentration of each types of studied CTCs after completion of immunomagnetic protocol described in Section 4.1. The SERS signal is generated from *p*-MBA Raman reporter molecules present in the SERS-immune probes. Figure 5 shows the averages SERS spectra in the presence of various analyzed cancer cells concentration (from 0–100 cancer cells/mL) in 1 mL of blood plasma. As we can see, the higher concentration of cancer cells results in stronger SERS response of immune system. The intensity of the selected marker band of Raman reporter at 1075 cm^−1^ linearly increased with the increase number of CTCs in blood sample (from 0 to 100 CTCs in 1 mL of blood). It should be noticed, that both bands 1075 cm^−1^ and 1587 cm^−1^ are characteristic for *p*-MBA and might be applied to construct the calibration curves. However, considering the 1500–1700 cm^−1^ range of biological subjects is reach in spectral fingerprints, the marker band at 1075 cm^−1^ of *p*-MBA has been selected for the quantitative analysis.

The error bars indicate the standard deviation from 15 different spots for each CTCs concentration. The blank spectrum was obtained for the blank plasma samples without adding cancer cells. In this case, the anti-EpCAM-Ag@Fe_2_O_3_/*p*-MBA were easily removed from anti-EpCAM-ALA-Ag/FLs substrate. However, it should be highlighted, that we cannot completely exclude their nonspecific adsorption without any immune recognition and some weak marker bounds may appear especially in the case of non-rigorous washing of immune–sandwich system with PBS at the last step of protocol (see Figure 2).

Subsequently, the low detection limits (LODs) were also determined using the signal-to-noise method on the basis of three standard deviation from the blank [105]. The linear relationships with R^2^ in the range from 0.82 to 0.98 demonstrate the potential of our method for quantitative analysis of cancer cell in blood plasma. Based in these data the detection limit was estimated as 1 CTC for LNCaP, 3 CTCs for PC3, 4 CTCs for A549, and 0 CTCs for HeLa in 1 mL of blood plasma.

It should be highlighted that the SERS response in the form of intensity of marker band at 1075 cm^−1^ is related with the level of EpCAM expression estimated via the Western Blot method. The experimental results, summarized in Table 1, prove the ability if our developed SERS-immune assay to complete the highly sensitive and quantitative analysis of CTCs even for CTCs with low EpCAM affinity.

This indicates that more Raman reporter-labeled–immune-magnetic nanoparticles were bound to LNCaP and PC3 than to A549 cells. This might be attributed the higher EpCAM expression and/or affinity on the LNCaP and PC3 than to A549 cell limes. According to our studies the HeLa cells are non-EpCAM expressing tumor cells and therefore are not detected in SERS-based immunomagnetic assay by immune-selection. The fact that not all tumor cells express EpCAM may generate a problem in a percentage of false negative results, thus number of CTCs might not be reliably assessed in clinical trials. On the other hand, our results showed that developed SERS method offers the reliable CTCs detection even despite low EpCAM expression in real clinical tumors samples. Our assay demonstrate the capability to detect circulating tumor cells from blood samples over a broad linear range (from 1 to 100 cells/mL) reflecting clinically relevant of CTCs amount depending on the stage of metastasis, age, applied therapy.

We have examined the lung tumor cell line and tumor samples from patient and revealed that the SERS response in our optofluidic device correlates with the level of EpCAM expression established by Western Blot analysis supported by immunocytochemistry.

In the present work, we showed that the efficiency of cancer cells numeration in the developed SERS-based immunomagnetic device depends on the level of EpCAM expression.

### 4.6. SERS Immunomagnetic Detection of CTC in Blood from Patients with Metastatic Lung Cancer

**Figure 6:**
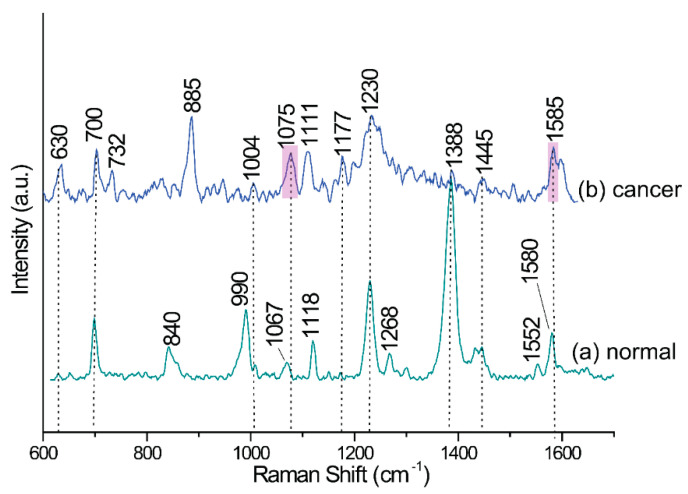
(**a**) SERS spectrum of the blood plasma of healthy volunteers (*n* = 5) and (**b**) SERS spectrum of metastatic lung cancer patients (*n* = 5) after applying the developed magnetically supported SERS-based immunoassay for the detection of circulating tumor cells.

Blood samples from lung cancer patients were used to validate the efficiency of the developed method in clinical trials. The number of CTC in the blood was analyzed in the samples from five metastatic lung cancer patients and compared to five samples from healthy volunteers. The constructed calibration curve for A549 was employed to measure the CTCs concentrations in clinical samples. As can be seen in Figure 6, both bands (1075 and 1587 cm^−1^) characteristic for *p*-MBA are evident for blood from metastatic lung cancer patients but not for peripheral blood samples from healthy patients. In the case of the clinical subject, the spectroscopic range of 1500–1700 cm^−1^ is located in spectral fingerprints corresponding to Amide I, Tryptophan, DNA/RNA, and others. In Figure 6 the band at 1587 cm^−1^ of *p*-MBA appears as a shoulder of wide band at 1580 cm^−1^, related to DNA/RNA bases in blood plasma of healthy subject [106], therefore the marker band at 1075 cm^−1^ has been selected for further quantitative analysis.

Table 2 presents the CTCs concentrations in healthy and cancer’ blood samples measured by SERS-immunoassay.

Compared with the blood from healthy people, SERS signals were found in blood of patients indicating the existence of CTCs. The concentration of CTCs in the clinical blood was measured to be from 5 to 13 cells in every 5 mL of blood (Table 2) according to the standard curve presented in Figure 4b. In one sample of the blood from a healthy person we have detected three circulating tumor cells. It might be related with the nonspecific adsorption of the immunomagnetic nanoparticles onto Ag/FLs SERS-active support without immune recognition or the tested blood sample was nevertheless from a patient with cancerous lesions.

In each case, it is extremely important to strictly follow the presented protocol of detection, and in particular to rigorously monitor the process of assay washing in the detection zone chamber.

## 5. Conclusions

In this work, we presented for the first time a magnetically assisted SERS-based immunoassay based onto solid SERS-active platform for selective isolation of four types of cancer cells and their non-invasive quantitative analysis in blood samples. We have examined the four different tumor cell lines and tumor samples from patient and revealed that the SERS response in our optofluidic device correlates with the level of EpCAM expression established by immunocytochemical analysis. Analysis of EpCAM expression by the Western Blot method supported by immunochemistry are consistent with the efficiency of SERS detection, which is inherent to this method as only EpCAM expressing cells are caughed from blood by immune-selection. These results are important for all methods which relay the expression of surface proteins and may give a false impression of negative results, as the level of EpCAM expression often shows variations.

The developed SERS-immunomagnetic assay was able to detect as low as five tumor cells in 5 mL of blood and successfully identified CTCs in metastatic lung cancer patients (positive results). The negative results were observed from healthy volunteer blood, which additionally validated the clinical potential of developed assay.

For future use of the developed approach in practical clinical analysis, the standardization of the whole procedure from biological samples sourced, conditions storage, and their preparation for SERS-based immune analysis to standardized SERS measurements conditions (e.g., type of excitation laser and time of spectra acquisitions) should be preserved.

## Figures and Tables

**Table 1:** Data obtained in SERS immune assay in comparison to Western Blot results.

Type of Cancer Cell	LOD Calculated via Immune SERS Assay	Intensity of Marker Band at 1078 cm^−1^ for 100 CTC in Blood Samples in cps (Count per Second) for 100 CTCs in 1 mL of Blood	Relative EpCAM Expression Estimated via Western Blot Method, See Figure 1A
LNCaP	1	9453	1.000
PC3	3	3612	0.320
A549	4	2570	0.014
HeLA	0	140	0

**Table 2:** Detection sensitivity of developed SERS immunoassay for clinical samples.

SERS Immune Assay Sensitivity (CTC in 5 mL of Blood Plasma)		
Sample Number	Metastatic Lung Cancer Patients (5 Samples)	Healthy Patients (5 Samples)
Sample #1	13	0
Sample #2	6	0
Sample #3	8	3
Sample #4	5	0
Sample #5	6	0

## Supplementary Materials

The following are available online at https://www.mdpi.com/2072-6694/12/11/3315/s1, Figure S1: Scheme of the magnetically controlled SERS-based microfluidic setup, Figure S2: The morphology of SERS-active substrates (Ag/FLS) were visualized by the scanning electron microscopy (SEM), Figure S3: SERS spectra of *p*-MBA (10^−6^ M) adsorbed on the surface of the platform, recorded for the Ag/FLS substrates, Figure S4: The real photo of the microfluidic system connected with syringe pumps, Figure S5: Supplementary data for the Western blot analysis shown in Figure 1 in the main text, Table S1: Summarizes band assignments for the normal Raman and SERS spectrum of *p*-MBA.
