# Supplementary material for: Effect of Varying Expression of EpCAM on the Efficiency of CTCs Detection by SERS-Based Immunomagnetic Optofluidic Device
Source: Cancers (Basel). 2020 Nov 10;12(11):3315. doi: 10.3390/cancers12113315 (PMC7697545; doi:10.3390/cancers12113315)
Supplement: Supplementary file 1 [file cancers-12-03315-s001.pdf]

Supplementary Materials

Effect of Varying Expression of EpCAM on the Efficiency of CTCs Detection by SERS-Based Immunomagnetic Optofluidic Device

Marta Czaplicka, Krzysztof Niciński, Ariadna Nowicka, Tomasz Szymborski, Izabella Chmielewska, Joanna Trzcińska-Danielewicz, Agnieszka Girstun and Agnieszka Kamińska

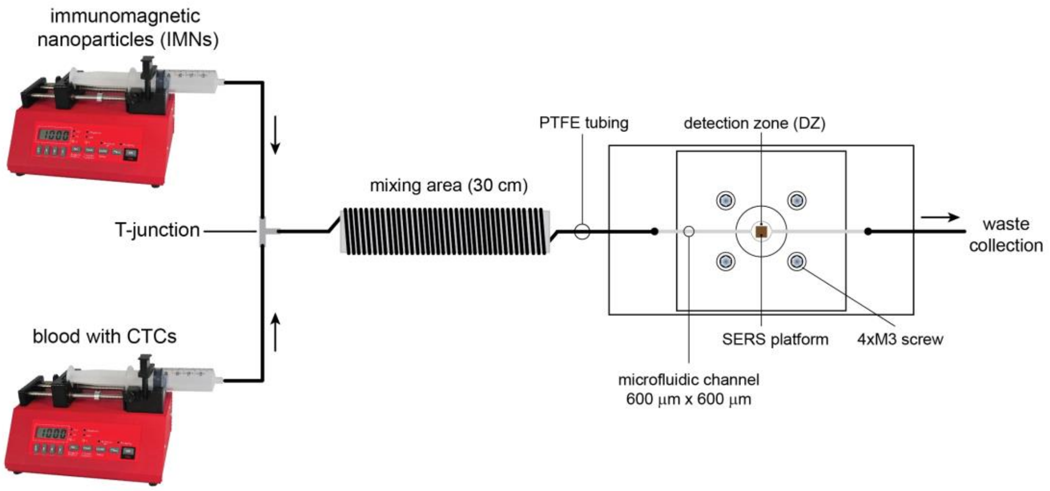

Figure S1. Scheme of the magnetically controlled SERS-based microfluidic setup.

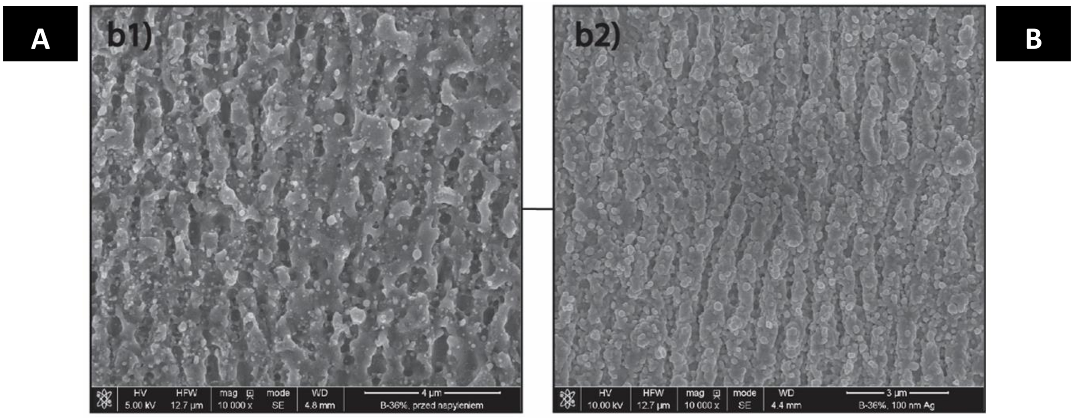

Figure S2. The morphology of SERS-active substrates (Ag/FLS) were visualized by the scanning electron microscopy (SEM). (**A**) surface of the silicon after laser ablation, (**B**) after laser ablation and sputtering with 100 nm of silver via PVD process.

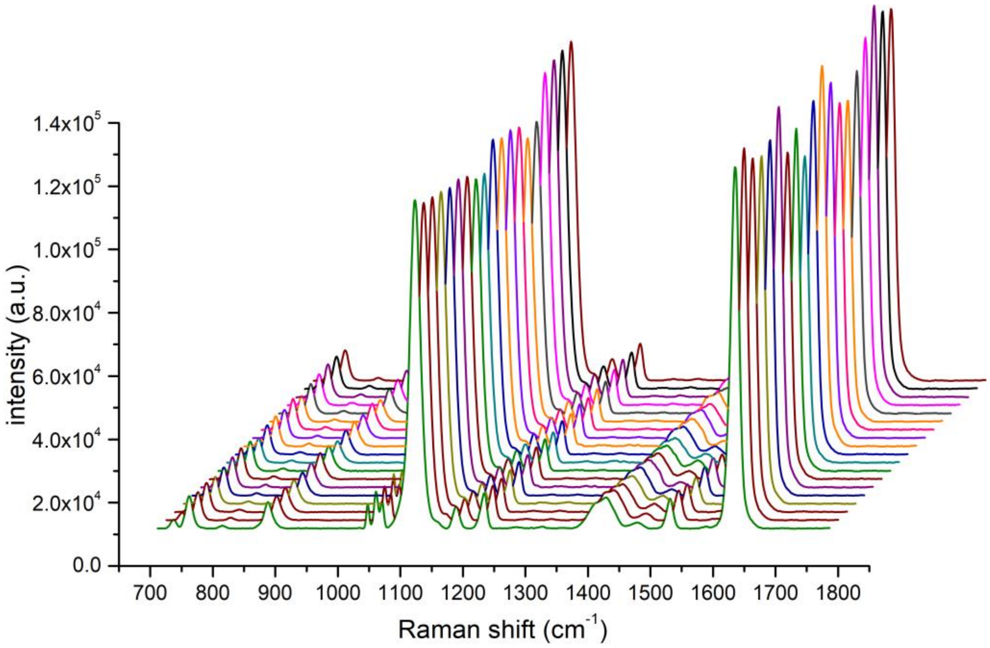

Figure S3. SERS spectra of *p*-MBA (10^{-6} M) adsorbed on the surface of the platform, recorded for the Ag/FLS substrates.

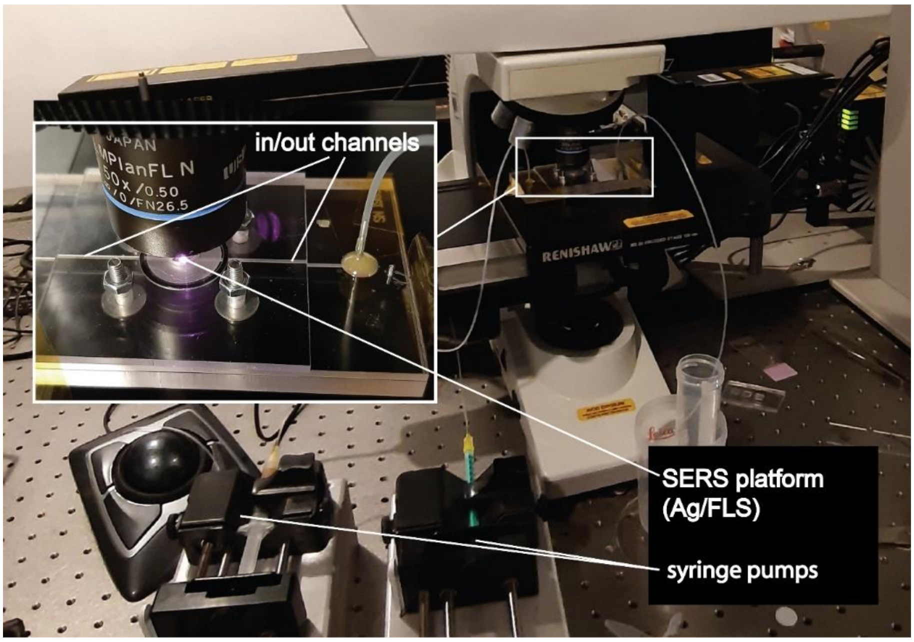

Figure S4. The real photo of the microfluidic system connected with syringe pumps.

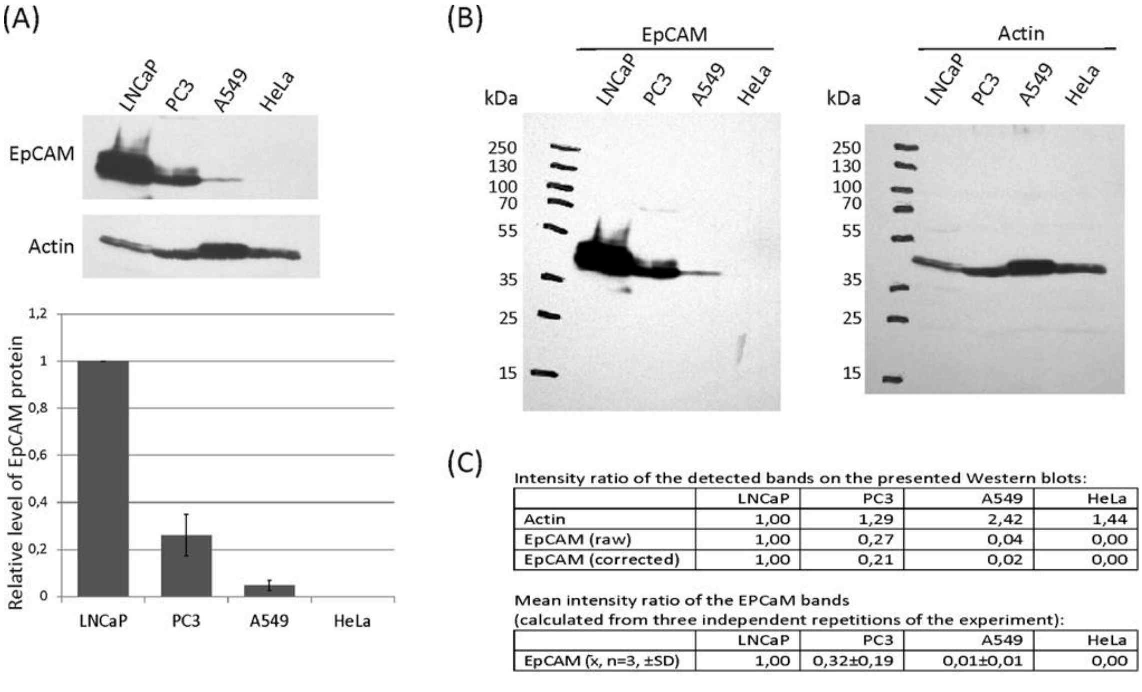

Figure S5. Supplementary data for the Western blot analysis shown in Figure 1 in the main text. (**A**) The image and graph presented in Figure 1A (details in the caption to Figure 1 in the main text). (**B**) The uncropped version of the Western blots shown in Figure 1A (the additional photos of the same films as in Figure 1A). The position of the molecular weight standards was marked with a pen on the film after Western blot detection according to the location of the prestained protein ladder (Thermo Scientific PageRuler Plus Prestained Protein Ladder) transferred along with cell lysates to the PVDF membrane. (**C**) The intensity ratio of the EpCAM bands in the probes analyzed on the presented Western blot (upper table) based on the densitometric quantification. The relative expression of EpCAM protein was calculated in reference to the level of EpCAM in LNCaP cells (arbitrarily set to 1). Actin level was used as a loading control. "EpCAM (raw)" corresponds to the direct quantification of the Western Blot densitometry readings for EpCAM; "EpCAM (corrected)" – data recalculated according to the measured actin level to correct the differences in the amount of loaded cell lysates. The same quantification and calculation were performed for two additional repetitions of experiments and mean values are presented in the lower table (data corrected according to the actin level).

Table S1. Summarizes band assignments for the normal Raman and SERS spectrum of *p*-MBA.

Normal Raman/*P*MBA Powder	SERS/*P*MBA onto Femtosecond Laser Induced SERS-Active Silicon Covered with 100 nm Layer of Silver	Assignments
1070	1075	$\upsilon(CC)_{ring}$
-	1143	$\upsilon(CCOO^{-})+$ $\upsilon(CS)$
1180	1187	δ (CH)
1357	1378	$\upsilon_s(COO^{-})$
-	1485	$\upsilon(COO^{-})$
1594	1589	$\upsilon(CC)_{ring}$

© 2020 by the authors. Licensee MDPI, Basel, Switzerland. This article is an open access article distributed under the terms and conditions of the Creative Commons Attribution (CC BY) license (http://creativecommons.org/licenses/by/4.0/).
